# SARS-CoV-2 infection augments species- and age-specific predispositions in cotton rats

**DOI:** 10.1038/s41598-022-27328-y

**Published:** 2023-01-14

**Authors:** Marina S. Boukhvalova, Emma Mortensen, Jessica Caple, John Joseph, Fatoumata Sylla, Arash Kamali, Daniel Stylos, Diego Lopez, Thomas March, Kevin Matthew Byrd, Gregory A. Prince, Ariel Arndt, Adriana Kajon, Jorge C. G. Blanco

**Affiliations:** 1 Sigmovir Biosystems, Inc., 9610 Medical Center Drive, Suite 100, Rockville, MD 20850 USA; 2 The ADA Science and Research Institute, LLC Chicago, Chicago, IL 60610-2678 USA; 3 Soft Cell Biological Research, Inc., St. George, UT 84770 USA; 4 Lovelace Biomedical Research Institute, Albuquerque, NM 87108 USA

**Keywords:** Viral infection, Experimental models of disease

## Abstract

Heterogeneity of COVID-19 manifestations in human population is vast, for reasons unknown. Cotton rats are a clinically relevant small animal model of human respiratory viral infections. Here, we demonstrate for the first time that SARS-CoV-2 infection in cotton rats affects multiple organs and systems, targeting species- and age-specific biological processes. Infection of *S. fulviventer,* which developed a neutralizing antibody response and were more susceptible to SARS-CoV-2 replication in the upper respiratory tract, was accompanied by hyperplasia of lacrimal drainage-associated lymphoid tissue (LDALT), a first known report of mucosa-associated lymphoid tissue activation at the portal of SARS-CoV-2 entry. Although less permissive to viral replication, *S. hispidus* showed hyperplasia of bone marrow in the facial bones and increased pulmonary thrombosis in aged males. Augmentation of these features by SARS-CoV-2 infection suggests a virus-induced breach in regulatory mechanisms which could be devastating for people of all ages with underlying conditions and in particular for elderly with a multitude of ongoing disorders.

## Introduction

The COVID-19 pandemic, caused by Severe Acute Respiratory Syndrome Coronavirus-2 (SARS-CoV-2), has affected the world since 2019, with 649,752,806 confirmed cases and 6,648,457 deaths estimated by the World Health Organization as of December 20, 2022^1^, though many more are suspected to have been infected or succumbed to the disease. While SARS-CoV-2 is a novel member of the genus *betacoronavirus* in the family Coronaviridae, two other members also present a serious health risk to humans, including SARS-CoV-1 which causes Severe Acute Respiratory Syndrome (9.5% fatality rate) and Middle East Respiratory Syndrome coronavirus (MERS-CoV, 34.4% fatality rate)^2,3^. Although SARS-CoV-2 has an overall lower case fatality rate: 0.1–5.8% in 20 of the most affected countries^4^, it has exhibited more efficient viral transmission.

The clinical course of COVID-19 ranges from asymptomatic to severe, with multi-organ failure that may lead to death. However, the most common symptomatology associated with COVID-19 resembles flu, including fever and dry cough^5–10^. The recent emergence of the highly transmissible omicron variant of coronavirus suggests that an even milder form of infection, often passing under the radar of public health surveillance, is possible^11^. Whether or not SARS-CoV-2 infection results in severe disease is dependent on a variety of known factors, including age, genetics, underlying comorbidities, and co-infections^12,13^, although other factors remain to be determined. Most hospital admissions involve SARS-CoV-2-infected patients who have co-morbid conditions, such as obesity, diabetes, hypertension, cardiovascular disease, or gastrointestinal disease^12^. Because of the wide range of disease manifestations and modifying comorbidities, modeling SARS-CoV-2 pathogenesis in animal models has been challenging. Several animal models of COVID-19 have been developed, including hamsters, mice, ferrets, and non-human primates (NHP), primarily for testing vaccines and therapeutics against COVID-19^14^. The course of SARS-CoV-2 infection in these animals, modeled without underlying conditions, ranges from strong lung involvement with severe pneumonia in golden hamsters to mild infections limited primarily to the upper respiratory tract in some NHP^14,15^. While no rat models have been published for COVID-19 to date, the cotton rat *Sigmodon hispidus* (SH) is an established clinically relevant model of human respiratory viral infections^16–20^. Most of the work has been performed in SH, however, there are multiple cotton rat species. For example, *Sigmodon fulviventer* (SF) is a less frequently used species of cotton rats, but it shows differences compared to SH in responses to human parainfluenza virus^21^ and respiratory syncytial virus (RSV) infections^22^, has a different microbiome community structure^23^, and appears to be more susceptible to infection with HCoV-229E (ongoing studies). These species-level differences may provide an opportunity to study the heterogeneity of the COVID-19 pathogenesis.

It remains unclear why there is such a wide range of clinical manifestations of COVID-19, how underlying conditions affect COVID-19 pathogenesis, and why only some individuals develop severe disease. It is also unknown how asymptomatic infections can induce strong immunity and protect against re-infection while vaccination is often accompanied by breakthrough infections. In this work, we challenged SH and SF with SARS-CoV-2 and compared their responses by considering age, sex, and diabetes as comorbidities. Our results establish cotton rats as an important model for COVID-19 considering age, sex, and comorbidities and highlight how species-specific differences in cotton rats’ infection susceptibilities may be of unique advantage in studying COVID-19 disease heterogeneity, re-infection susceptibilities, and variable outcomes of vaccination.

## Results

### SARS-CoV-2 infection in SH and SF

**Figure 1:**
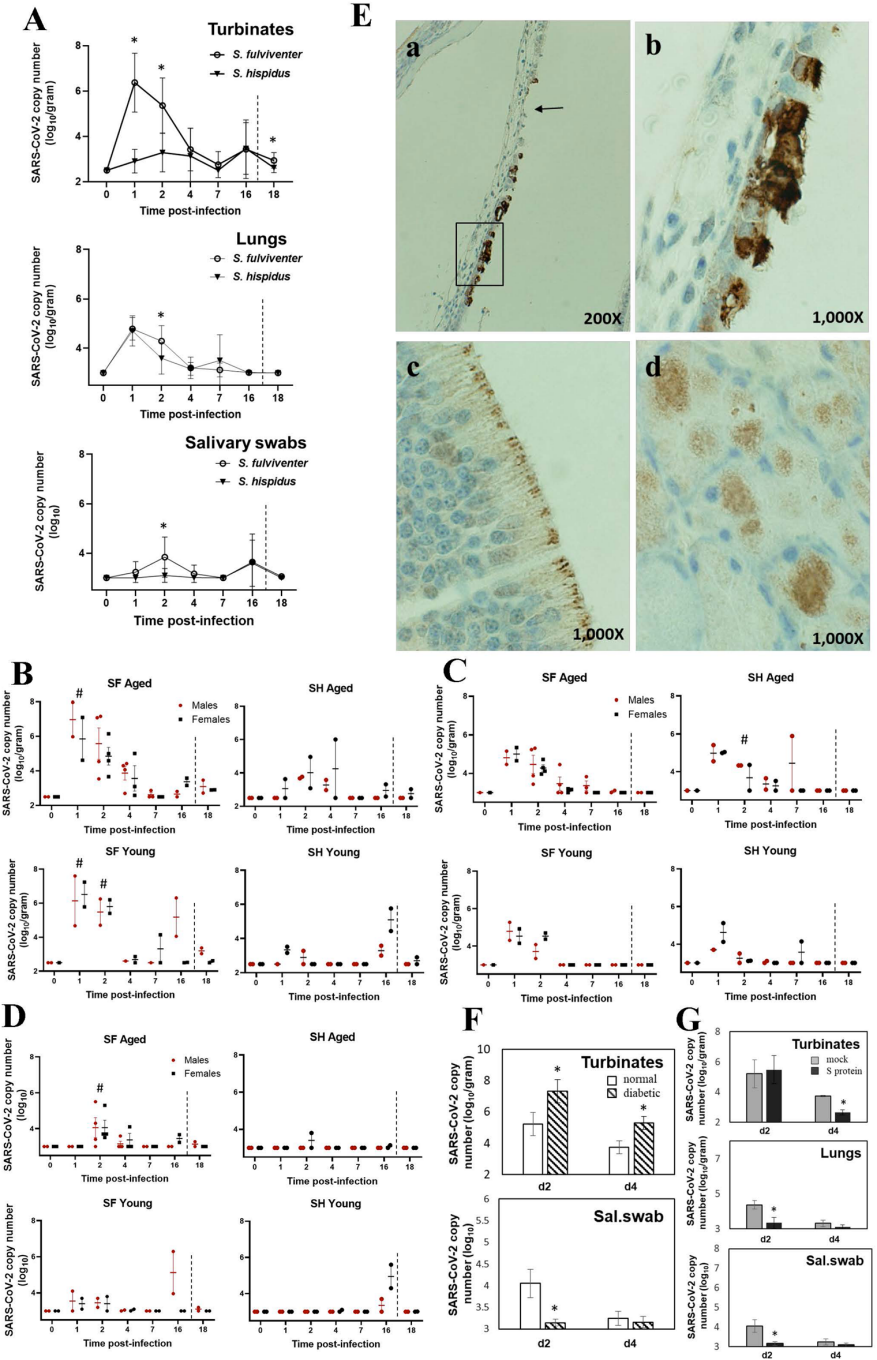
Viral replication in SARS-CoV-2-infected cotton rats. (**A**) *S. fulviventer* (SF) and *S. hispidus* (SH) were inoculated with SARS-CoV-2 and sacrificed on days 1–16 post-infection for analysis of viral load in turbinates, lungs, and salivary swabs by qPCR. A group of animals was re-challenged with SARS-CoV-2 on day 16 post-infection and sacrificed 2 days later (day 18, dashed line indicates re-infection). The data for males and females of different ages are presented altogether. Seven to twelve animals per time point. *p < 0.05 compared to SH*.* (**B**–**D**) Viral loads shown separately for male or female, aged or young animals for turbinates (**B**), lungs (**C**) or salivary swabs (**D**). The mean ± SE is shown for each time point. #*p* < 0.05 compared to SH of the same age category (males and females together). (**E**) Nasal sections of SF infected with SARS-CoV-2 and sacrificed on day 1 post-infection were stained for SARS-CoV-2 S protein. Virus was detectable in the respiratory epithelium of maxilloturbinate (a,b). Panel a shows damaged area of respiratory epithelium (arrow) that already does not contain viral antigens and another area of epithelium (boxed) intensely stained that is further magnified in panel b. Olfactory epithelium (c) and submucosal glands surrounding the maxillary sinus (d) were also positive for SARS-CoV-2. (**F**) SF with diabetes and normal SF were challenged with SARS-CoV-2 and sacrificed on days 2 and 4 post-challenge for analysis of viral load in the turbinates and salivary (Sal.) swabs by qPCR. The mean ± SE is shown for each group of cotton rats (4–8 animals per group, males and females). *p < 0.05 compared to normal animals. (**G**) SF were vaccinated with the purified S protein of SARS-CoV-2 adjuvanted with alum, boosted, and infected with SARS-CoV-2. Control animals were mock-immunized with alum, infected with SARS-CoV-2, and sacrificed in parallel with the S-protein-immunized infected animals on days 2 and 4 post-infection for analysis of viral load in turbinates, lungs, and salivary swabs by qPCR. Results represent the mean ± SE for each group of animals (4–8 animals per group, males and females). *p < 0.05 compared to mock-immunized, SARS-CoV-2-infected animals, sacrificed on the same day.

Two cotton rat species, SH and SF, were challenged with SARS-CoV-2 intranasally and ocularly for the first time. Viral load was evaluated in nasal turbinates, lungs, and salivary swabs of infected animals sacrificed on days 1–16 post-infection (Fig. 1A). A group of infected animals was re-challenged with SARS-CoV-2 16 days after the initial infection and sacrificed 2 days later for analysis of secondary infection (time point d18).

The highest level of SARS-CoV-2 was present in the turbinates of SF. Lower levels of SARS-CoV-2 were present in the lungs and salivary swabs, with differences between species being less pronounced. A detailed analysis of age- and sex-dependent outcomes in infected animals showed that the maximum level of virus in the turbinates of aged and young SF on days 1–2 post-infection was comparable, but there was more virus in the turbinates of aged SF on day 4 post-infection (Fig. 1B). In young SF, the virus peaked on days 1–2, went back to baseline on day 4, and then reappeared in select animals on days 7–16 post-infection. Virus reappeared during the second week of infection in select SH as well. Re-challenged SH or SF (day 18, young and old) did not show any increase in viral load compared to the time of re-challenge (day 16), indicating that immunity to re-infection was established within 16 days of primary challenge.

Detection of SARS-CoV-2 in the lung was maximal on days 1–2 post-infection in both species (Fig. 1C). Comparable levels of pulmonary SARS-CoV-2 load were seen for SF and SH on day 1, with slightly more virus detected in SF on day 2 post-infection. Prolonged viral presence was detected in the lungs of aged SF. A delayed or rebound of viral replication was seen in select aged and young SH one week after infection. No virus was detected in the lungs of either SF or SH re-infected on day 16 and sacrificed 2 days later (day 18).

Saliva is now a viable alternative to nasopharyngeal swabs as a test for COVID-19^24^ and may be able to transmit SARS-CoV-2^25^. Relatively low levels of virus were detected on days 2–4 in swabs from aged SF and on day 2 in one aged SH (Fig. 1D). Young SF had virus in the swabs collected on days 1–2 and then on day 16, while young SH showed virus in salivary swabs only on day 16. Immunohistochemistry of nasal/paranasal tissues using antibodies specific for SARS-CoV-2 S protein revealed the presence of viral antigens in the respiratory and olfactory epithelium as well as in submucosal glands (Fig. 1E). Respiratory epithelium positive for S protein was located primarily in the lining of maxillary turbinates (Fig. 1E a, b). No IHC was carried out for the lungs.

As diabetes presents a risk factor for COVID-19, a diabetic cotton rat model was established and used in this work. Diabetic cotton rats SF infected with SARS-CoV-2 showed significantly more virus in the turbinates on days 2 and 4 post-infection (Fig. 1F). The amount of virus in the lungs of diabetic and non-diabetic SF was comparable. The amount of SARS-CoV-2 detected in salivary swab samples of diabetic animals, however, was significantly less than that detected in non-diabetic animals on day 2 post-infection (Fig. 1F).

Vaccination with S-protein of SARS-CoV-2 was evaluated for its ability to protect cotton rats against SARS-CoV-2 infection. Viral load in turbinates of S-protein-vaccinated animals was indistinguishable from that in mock-vaccinated animals two days after SARS-CoV-2 infection (Fig. 1G). On day 4 post-infection, however, significantly less SARS-CoV-2 was detected in turbinates of vaccinated cotton rats. In the lungs, vaccination significantly reduced viral load on day 2 post-infection. The same trend was reflected in salivary swabs (Fig. 1G).

### Antibody responses to SARS-CoV-2 infection

**Figure 2:**
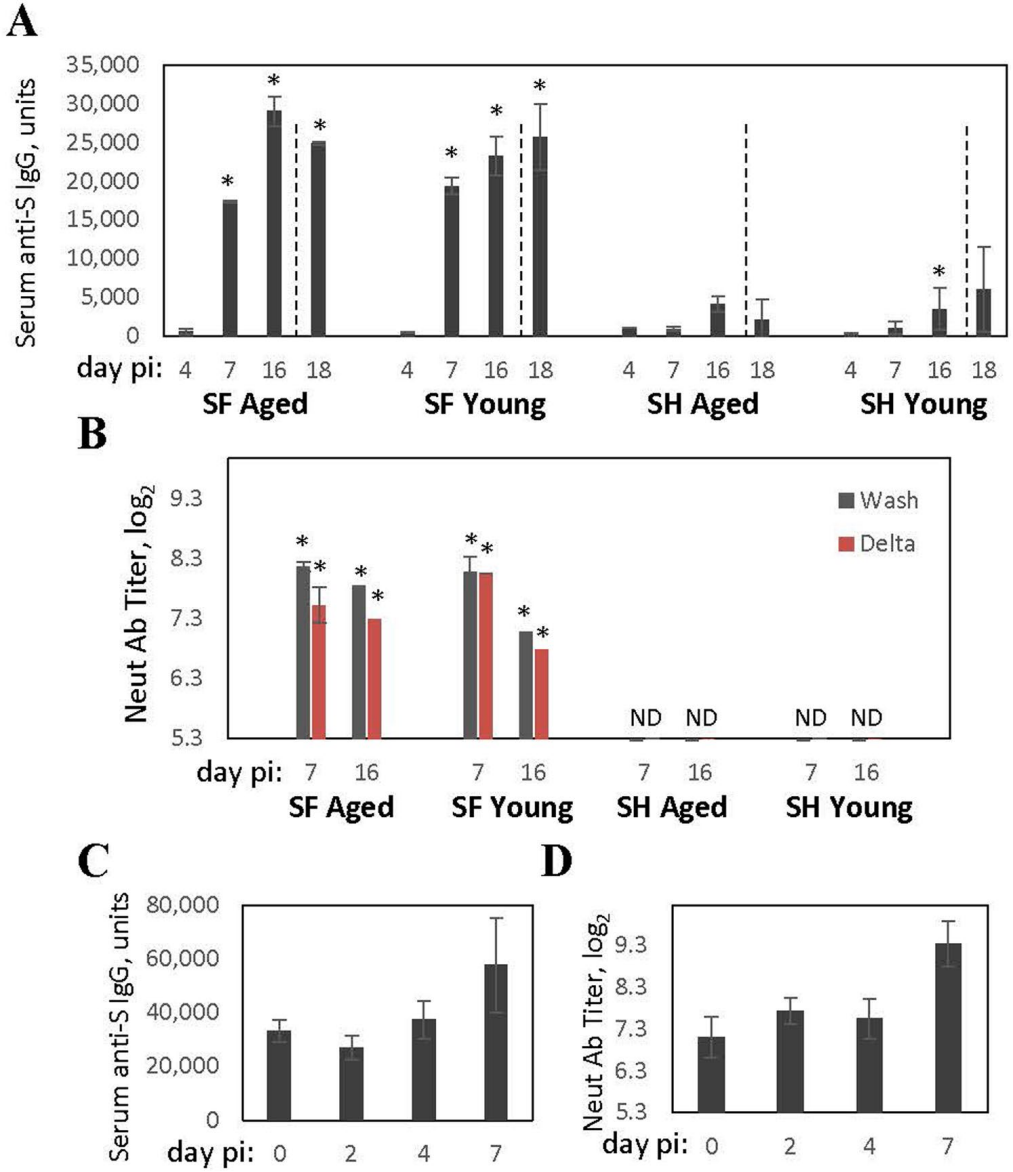
Antibody response of cotton rats to SARS-CoV-2-infection. (**A**) Aged and young cotton rats SF and SH were infected with SARS-CoV-2, and sera was collected for analysis of binding IgG against SARS-CoV-2 S protein on days 4, 7, and 16 after infection. A group of animals was re-challenged with SARS-CoV-2 on day 16 post-infection and sera was collected 2 days later (day 18, dashed line indicates re-infection). (**B**) Neutralizing antibodies in the serum of aged and young infected SF or SH on days 7 and 16 post-infection. Neutralization was measured against homologous (Washington, Wash, dark grey bars) and heterologous (Delta, red bars) strains of SARS-CoV-2. No neutralizing antibody activity was detected against either virus in sera collected from SARS-CoV-2 infected SH (ND). Results represent the mean ± SE shown for each group (males and females). *p < 0.05 compared to uninfected animals. (**C**) Binding IgG and (**D**) neutralizing antibodies against Washington strain in sera of cotton rats immunized with S protein and sampled immediately prior to SARS-CoV-2 infection (day 0) or on days 2, 4, and 7 after SARS-CoV-2 infection.

SF infected with SARS-CoV-2 developed rapid S-binding IgG and neutralizing antibody responses to infection (Fig. 2). S-binding IgG level (Fig. 2A) increased significantly between days 4 and 7 post-infection and was comparable on day 7 in aged and young SF. A further increase in S-binding IgG was seen between day 7 and day 16 post-infection for both age groups. No further increase was detected in re-challenged aged animals two days after repeat exposure. S-binding IgG response in infected SH was lower than in infected SF, with a significant increase in antibody levels visible only on day 16 post-infection.

A strong neutralizing antibody response was detected in infected SF one week after infection (Fig. 2B), with neutralization against both homologous (Washington) and heterologous (Delta virus strain USA-GNL-1205/2021) SARS-CoV-2 viruses detected in both aged and young animals. No neutralizing antibodies against SARS-CoV-2 were detected in SH (Fig. 2B). Neutralizing antibody response in diabetic SF one week after infection was comparable to that in non-diabetic animals (7.79 ± 0.22 Log_2_ in diabetic vs. 8.19 ± 0.39 Log_2_ in non-diabetic SF).

S-protein vaccination induced strong S-binding IgG and neutralizing antibody responses in SF (Fig. 2C,D, day 0). Anti-S IgG level decreased slightly 2 days after SARS-CoV-2 infection, and then increased by day 7 after challenge (Fig. 2C). Neutralizing antibody level in vaccinated SF increased from 7.13 ± 0.5 to 9.37 ± 0.6 Log_2_ within a week of challenge (Fig. 2D).

### Nasal and bone marrow histopathology in SARS-CoV-2-infected animals

Histology of nasal, paranasal, and oral tissues was examined in head samples from cotton rats SF and SH after SARS-CoV-2 infection. Rhinitis was present in a few specimens where small foci of olfactory, transitional, or respiratory epithelium were minimally disrupted, degenerated, or hyperplastic in response to infection, and the underlying lamina propria was infiltrated by a small number of mostly mononuclear leukocytes with fewer granulocytes. Rhinitis was not a prevalent lesion, but the amount of nasal mucosal tissue for evaluation was often scant, fragmented, or otherwise missing in specimens prepared from bisected heads. More severe injury to the nasal mucosa could be missed with this evaluation.

**Figure 3:**
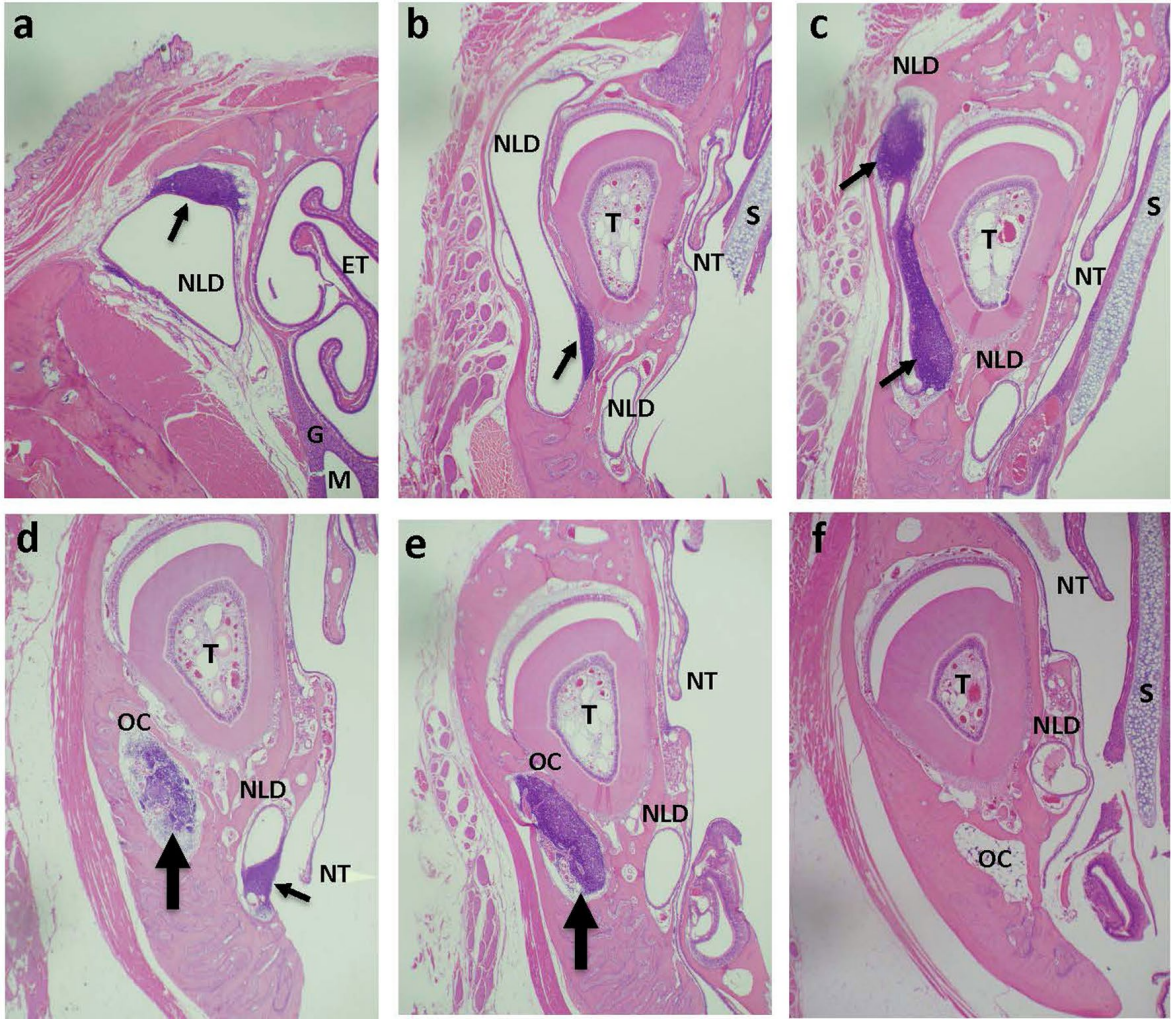
Lacrimal drainage-associated lymphoid tissue (LDALT) hyperplasia after SARS-CoV-2 infection in cotton rats SF. Nasal/paranasal sections from SARS-CoV-2-infected (**a**–**e**) or control uninfected (**f**) SF at the level of the second palatal ridge (**a**) or at the level of incisive papilla (**b**–**f**). (**a**) The caudal portion of nasolacrimal duct (NLD) of infected SF on day 7 post-infection showing LDALT (arrow) at the top corner of NLD. Ethmoid turbinates (ET), maxillary sinus (M), and submucosal glands (G) are visible. (**b**) The same animal as in panel a, but with the head cut at the level of incisive papilla. NLD is visible in two locations: lateral to the incisor tooth (T) and below it. LDALT is seen only in the portion of the duct lateral to the tooth (arrow). The septum (S) and nasoturbinates (NT) are visible in this sample. (**c**) An example of a more intense activation of LDALT (arrows) in a different SF on day 14 post-infection in a cut position similar to that shown in panel b. (**d**, **e**) Lymphoid hyperplasia leading to the “occlusion” (thick arrow) of the osseous canal (OC) housing NLD in infected SF on day 4 (**d**) or day 7 (**e**) post-infection. A ventral section of NLD shows signs of LDALT activation as well (**d**, thin arrow). (**f**) The section from a control, uninfected SF showing lack of LDALT hyperplasia and lack of occlusion of OC. H&E stain, 20X magnification.

The most notable change in the nasal/paranasal tissues of infected animals was hyperplasia of lacrimal drainage-associated lymphoid tissue (LDALT) in infected SF (Fig. 3). Infected animals demonstrated lymphoid hyperplasia (referred to as hyperplasia from now on) visible in different areas of the nasolacrimal duct (NLD) depending on the cut (Fig. 3a–e, Suppl Table 1). In some instances, hyperplastic lymphoid aggregates were seen in the osseous canal housing NLD (Fig. 3d,e). In addition to LDALT hyperplasia, many infected SF showed hyperplasia of nasal-associated lymphoid tissue (NALT) located at the nasopharyngeal opening (Suppl Table 1). NALT, however, was not visible in many of the samples, interfering with its full evaluation. Nevertheless, LDALT and NALT hyperplasia was more evident in SARS-CoV-2-infected SF than SH (Suppl Table 1). Hyperplasic changes in one or both structures were seen as early as day 1–2 post-infection, with the increase remaining evident for the following two weeks. Some LDALT and NALT hyperplasia was also seen in select control, uninfected SF. In contrast to SF, SH did not show LDALT hyperplasia. There was some hyperplasia of NALT in SH, but it was seen predominantly in young animals and detected mostly later in infection (days 7–16).

**Figure 4:**
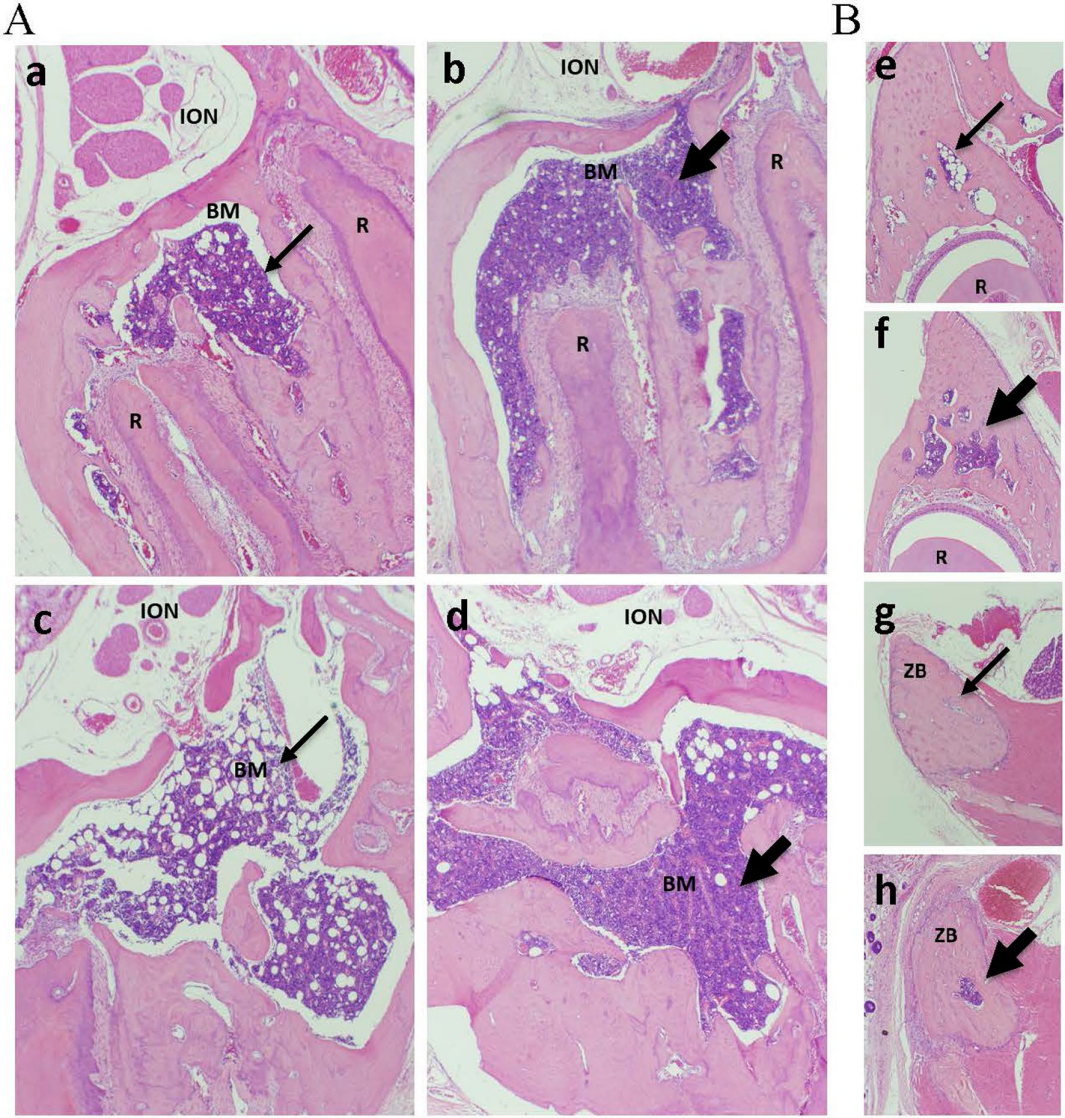
Bone marrow (BM) hyperplasia in SARS-CoV-2-infected cotton rats SH. (**A**) BM hyperplasia in maxilla at the molar tooth level cut. Young (a,b) and aged (c,d) SH males were mock-challenged with PBS (a,c) or infected with SARS-CoV-2 and sacrificed on day 7 (b) or day 4 (d) post-infection. BM, bone marrow; R, tooth root; ION, infraorbital nerve. (**B**) BM in additional facial bone areas: in maxilla at the incisor papilla level cut (e,f) and in the zygomatic bone (ZB) (g,h). Young SH male mock-challenged with PBS (e,g) or infected with SARS-CoV-2 and sacrificed 7 days later (f,h). Thick arrows point to hyperplastic BM, thin arrows point to normal BM. H&E, 40X.

In addition to changes in mucosa-associated lymphoid tissue, SARS-CoV-2 infection led to changes in bone marrow (BM). Hyperplastic BM with densely packed red and white precursors displacing stroma and adipocytes was seen in the facial bones of infected SH (Fig. 4, Suppl Table 1). Changes were most noticeable in young SH males and aged SH males and females, with the more prolonged changes in the latter (Suppl Table 1). An overabundance of myeloid cells was seen in the BM of infected animals. Myeloid to erythroid cell ratio was calculated for young SH males before infection and 7 days after infection and was found to increase from 1.08 in uninfected to 1.38 in SARS-CoV-2-infected animals. There was less BM tissue in the heads of SF compared to SH at baseline (uninfected) and infection-induced changes in the BM of SF were more subtle and/or more difficult to detect. In addition to these changes, inflammation was noted in the connective tissue in the vicinity of the infraorbital nerve at the tooth root of infected SF (Suppl Table 1). This effect, however, was seen only sporadically in infected animals (and one uninfected young SF animal), and the full extent of this observation is not clear.

### Pulmonary histopathology in SARS-CoV-2-infected animals

**Figure 5:**
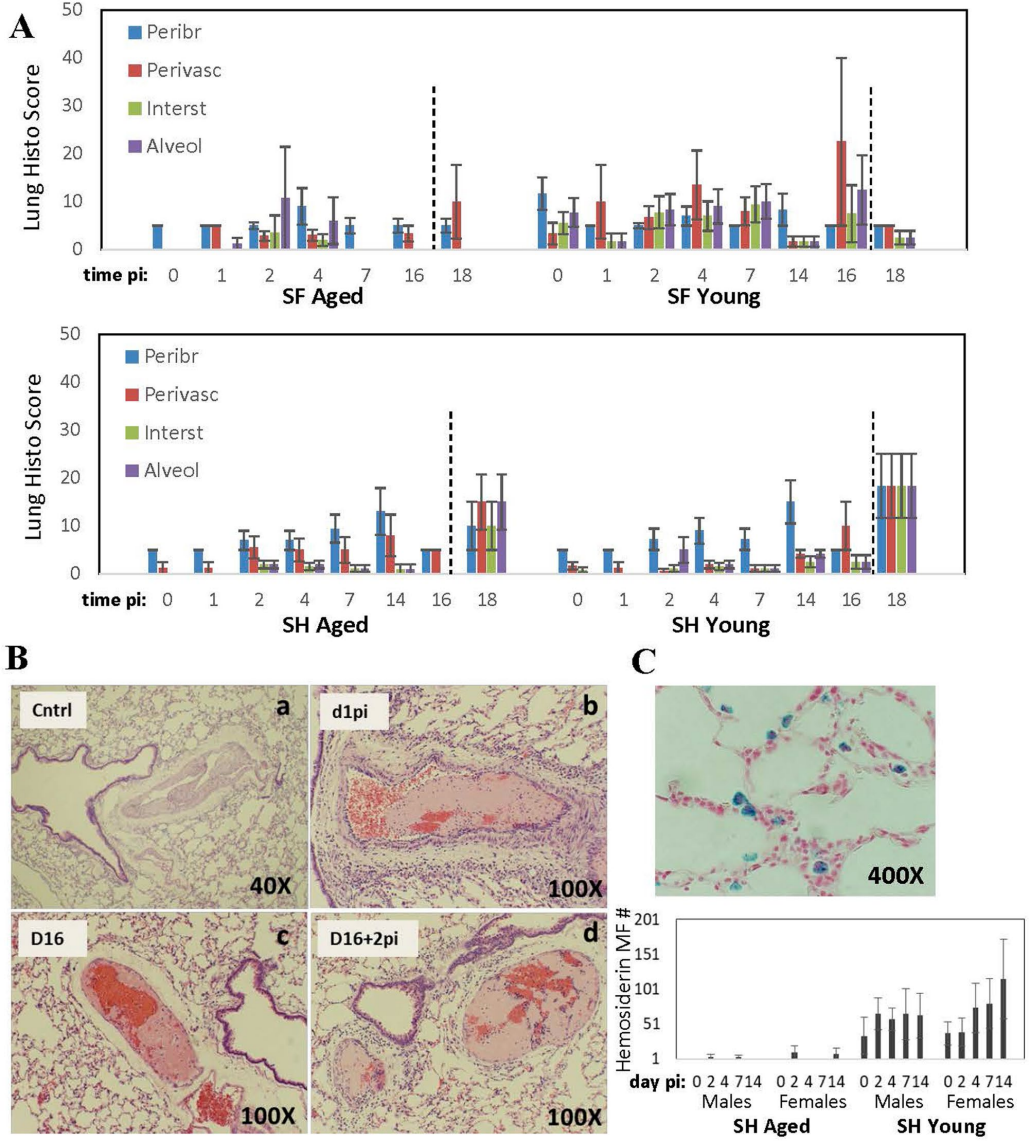
Pulmonary histopathology in cotton rats infected with SARS-CoV-2. (**A**) Peribronchiolitis (Peribr), perivasculitis (Perivasc), interstitial inflammation (Interst) and alveolitis (Alveol) were evaluated in H&E-stained lung samples from aged and young SF and SH infected with SARS-CoV-2 (or re-infected) as described in the legend to Fig. 1. Dashed line indicates re-infection. Results are cumulative of two independent studies (4–10 samples per time point, males and females). (**B**) Pulmonary thrombosis in aged male SH*.* (a) A hypertrophic blood vessel wall in uninfected (Cntrl) animal, (b–d) acute thrombi in SARS-CoV-2-infected animals on days 1, 16, and 16 + 2 (after re-infection). (**C**) Hemosiderin-positive macrophages in the lungs of young SH on day 14 post-infection (Prussian Blue stain) and graphic summary of positive cells in aged and young SH on various days after SARS-CoV-2 infection (no hemosiderin-positive macrophages were detected in SF)*.*

Pulmonary histopathology was assessed in SARS-CoV-2-infected animals in concordance with standard parameters evaluated in respiratory infection models using cotton rats, but showed only mild increases in response to SARS-CoV-2 infection in all groups of cotton rats examined (Fig. 5A). Overall extent of pulmonary inflammatory response appeared to be the strongest in young SF. However, this group of animals also showed the highest basal level of pulmonary inflammation in uninfected animals, suggesting a heightened inflammatory environment in the lungs of SF compared to SH.

Increased pulmonary thrombosis was seen in aged male SH (Fig. 5B, Suppl Table 2). Between 33 and 100% of lung samples of infected aged male SH sacrificed between day 1 and 16 post-infection (or re-challenged animals on day 18) demonstrated pulmonary thrombi (Suppl Table 2, Fig. 5B b–d). This effect could be sex-related, as none of the aged SH females had pulmonary thrombi, except for one aged female on day 7 post-infection (Suppl Table 2). Thickened blood vessel walls were seen in some control aged SH males (Fig. 5Ba). One mock-challenged aged SH male also had pulmonary thrombi, suggesting a natural propensity for blood vessel wall hyperplasia and thrombosis in this category of animals. Hemosiderin-positive macrophages were detected in the lungs of young SH, and their number increased with infection (Fig. 5C). Overall, these findings point to the complexity of pulmonary manifestations of SARS-CoV-2 beyond what has been previously shown for other respiratory viruses in the model.

## Discussion

Cotton rats are a clinically relevant small animal model for a variety of human respiratory viral infections^16–20^. In the current work, we infected cotton rats for the first time with SARS-CoV-2. Of the two cotton rat species tested, SF developed a neutralizing antibody response to SARS-CoV-2 infection and was more susceptible to SARS-CoV-2 replication in the upper respiratory tract. Both species had comparable limited viral replication in the lung, without strong inflammatory changes or signs of acute respiratory distress syndrome (ARDS) and did not display outward clinical signs in the form of weight loss or labored breathing. Infection of cotton rats SF and SH with SARS-CoV-2, thus, appears to resemble a mild form of SARS-CoV-2 infection rather than its severe pulmonary form. This contrasts with hamsters that develop severe interstitial pneumonia, with robust viral replication in the lungs^26^.

The cotton rat model seems to show some similarities to non-human primates, where SARS-CoV-2-induced disease can be milder^27^. Coincidentally, the first SARS-CoV-2 human challenge study showed that SARS-CoV-2 induces a mild-to-moderate or asymptomatic disease, with high viral load detected even in asymptomatic individuals^28^. The most frequently detected symptoms in that challenge study were related to the upper respiratory tract and included nasal stuffiness, rhinitis, sneezing, and sore throat^28^. Before this work, coronaviruses have not been tested extensively in cotton rats. One publication from 2008^29^ described challenging cotton rats with SARS-CoV-1 and reported a lack of weight loss, inconsistent viral detection in nasal washes, and no evidence of ARDS in SARS-CoV-1-infected animals. Despite the small number of animals used, however, neutralizing antibody response was found in infected SF and not SH^29^, suggesting a commonality in increased susceptibility of SF to SARS-CoV-1 infection as well.

The upper respiratory tract appears to be the main target of SARS-CoV-2 infection and replication in cotton rats. SARS-CoV-2 was detected in the nasal respiratory and olfactory epithelium as well as submucosal glands, particularly mucous acini. The respiratory epithelial cells infected by SARS-CoV-2 clustered in the lining of the maxillary turbinate, close to virus-positive submucosal glands, pointing to specific areas of increased susceptibility. The heterogeneity of cells in human nasal/oral mucosa and their variable susceptibility to SARS-CoV-2 infection based on patchy receptor expression clusters have recently been reported^25^. Mucous acini of minor salivary glands were found to harbor a low frequency of ACE2/TMPRSS2 co-expressing cells supporting their SARS-CoV-2 infection capabilities^25^. The finding of viral antigens in submucosal glands of infected cotton rats, particularly in the mucous acini clusters, may be representative of a similar heterogeneity of susceptible cells/sites in the cotton rat nasal cavity. SARS-CoV-2 was detected by qPCR in the turbinates of infected cotton rats within days of challenge and then again during the second week post-infection (day 16), suggesting persistent or rebound infection that has not been seen so far with other respiratory viruses in the model. Of interest, the recent SARS-CoV-2 human challenge study also showed a small increase/plateau in viral load by qPCR in nose and throat samples on days 16 and 18 post-infection, following preceding continuous decline in viral load^28^.

Immunity to re-infection was formed in cotton rats during the first two weeks of the initial exposure to the virus, protecting both SF and SH against viral infection in both the upper and lower respiratory tracts. In SF, immune response to SARS-CoV-2 infection included a strong increase in both serum IgG against S protein and SARS-CoV-2 neutralizing antibodies, while in SH the response was limited to a small upregulation in anti-S IgG and no detectable serum neutralizing antibodies. It is possible that the more efficient replication of virus in the nose of SF and the differences in the mucosa-associated lymphoid tissue organization/function between SF and SH may be responsible for the inefficient serum neutralizing antibody response development in SH as discussed below. In spite of these differences, the immunity induced by primary infection in either SF or SH was sufficient to protect animals against re-infection, suggesting that serum neutralizing antibodies may not be the main correlate of protection against SARS-CoV-2 infection. Similarly, high-level neutralizing antibodies induced in SF by intramuscular immunization with alum-adjuvanted S protein did not prevent infection of the nose, albeit the vaccination did facilitate faster nasal clearance and protected the lungs early on. These results agree with the findings in rhesus macaques, where SARS-CoV-2 vaccination does not alleviate nasal replication, but reduces lung involvement^30,31^ and could mimic those findings in humans, where even vaccinated individuals undergo repeated infections^32^. Alternative correlates of vaccine-induced protection against SARS-CoV-2 infection may be needed.

Diabetes is one of the most important factors in defining COVID-19 severity in humans and was considered one of the variables in our studies. SARS-CoV-2 replicated more efficiently in turbinates of diabetic SF compared to non-diabetic ones, suggesting that diabetes may cause increased susceptibility to infection. This was not accompanied by a higher replication of the virus in the lungs or increased lung pathology (not shown), possibly due to the restricted number of time points used for analysis. It is also possible that COVID-19 results in a more severe form in people with diabetes because of the additional aggravating conditions (e.g., obesity, renal problems, or hypertension) commonly associated with diabetes, that our diabetic model could not entirely replicate. Interestingly, while the amount of SARS-CoV-2 detected in turbinates of diabetic SF was significantly higher than in turbinates of normal animals, salivary swabs showed an opposite association, possibly due to hyposalivation and xerostomia commonly associated with diabetes^33^.

The effect of aging on SARS-CoV-2 pathogenesis was evaluated as a part of our studies. Both species of cotton rats demonstrated that the virus persisted longer in the respiratory tract of aged animals compared to the young ones, in line with the findings from other animal models of infection with SARS-CoV-2^34^ or mouse-adapted SARS-CoV-2^35^ and similar to findings with respiratory syncytial virus (RSV) in SH^36–38^. Similar to RSV or influenza in cotton rats^36,37,39^, no increase in pulmonary inflammation was seen in aged cotton rats infected with SARS-CoV-2 compared to young animals. Pulmonary thrombosis, however, accompanied infection in aged SH. The thrombosis was almost exclusively limited to the aged males, suggesting both age- and sex-specific preference. Some of the control, uninfected aged SH also had thrombi in the lungs. Blood vessel walls in the lungs of some aged SH males appeared hypertrophic at baseline. A physical impediment to blood flow due to vessel wall hypertrophy, especially under conditions of coagulopathy, could contribute to the development of thrombosis and embolism. Changes in blood coagulation and thrombotic manifestations are among the most reported features of human SARS-CoV-2 infection^40,41^.

“Silent” or “happy” hypoxia is frequently seen in COVID patients^42,43^. Many patients show reduced blood oxygen levels that some experts describe as “incompatible with life”, while lung scans show little to no abnormalities and patients do not display symptoms of shortness of breath or difficulty breathing^44^. Virus-induced changes in hematopoiesis, altered turnover of erythrocytes, and the presence of malfunctioning erythrocytes in the lungs may contribute to silent hypoxia and could be the key players in determining the severity of COVID illness^45,46^. Analysis of early events of SARS-CoV-2 infection in cotton rats suggests that SARS-CoV-2 infection may cause BM hyperplasia that is accompanied by a reduced proportion of erythroid cells in the BM of facial bones. The effect was more predominant in SH in spite of the less efficient nasal SARS-CoV-2 replication in SH compared to SF. It is possible that the interaction between viral molecules already present in the inoculum and Toll-like receptors (TLRs) in the nasal/oral mucosa is sufficient to initiate changes in hematopoiesis. Hematopoiesis is dependent on a variety of TLRs during infections^47^, and SARS-CoV-2 in particular was shown to activate TLR signaling in a replication-independent manner^48^. BM hyperplasia has been reported in COVID patients^49,50^. To the best of our knowledge, this is the first report of SARS-CoV-2-induced changes in the BM of facial bones in an animal model. These changes would be difficult to visualize in humans but may have important consequences. Human maxilla and mandibles contain a large amount of BM (23% and 16%, respectively)^51^ which could be a vast target for virus-induced changes. Recent studies have shown that the nose and lungs are important sources of hematopoietic stem cells (HCS)^52–54^. Modification of mechanisms regulating HCS development in the nose/facial bones may have long-lasting systemic consequences. Moreover, because of the local BM changes close to the portal of SARS-CoV-2 entry, systemic prophylaxis against infection may not be fully effective.

The work reported here demonstrated for the first time (to the best of our knowledge) that SARS-CoV-2 infection can lead to lymphoid hyperplasia of LDALT. The existence of the organized LDALT (sometimes also called Tear Duct-Associated Lymphoid Tissue (TALT)^55^) in humans has been described for the first time in 2001^56^. However, little is still known about LDALT, particularly the portion associated with NLD. Human NLD cannot be analyzed as a part of dacryocystectomy, and references must be drawn from animal models. Studies of animal LDALT, however, have also been limited due to the difficulties in obtaining decalcified head samples for histopathology analysis. SF demonstrated well-defined LDALT showing signs of lymphoid hyperplasia after SARS-CoV-2 infection, while SH did not. Some mock-infected SF also displayed minimal LDALT presence, suggesting that the LDALT system is active in normal mucosal surveillance in SF. The interspecies differences in detection of organized LDALT in SF and SH and differences in the extent of LDALT changes in the model may be important for intraspecie studies of the heterogeneity of LDALT in humans, where the follicular organization of LDALT is seen in ~ 28–50% of individuals^56,57^. It may also indicate that the diversity of immune responses to SARS-CoV-2 infection and different levels of immunity to infection could be due to anatomical/functional differences in LDALT. Processing of viral antigens through the LDALT (as in SF) may be required for the establishment of a neutralizing antibody response against SARS-CoV-2. Moreover, the acute lymphoproliferative response in the NLD may cause a drainage impediment into the nasal cavity and lead to conjuctival congestion and ocular abnormalities seen in COVID patients^6,58–60^.

One of the most interesting findings of this work was the fact that innate biologic parameters characteristic of a certain population of cotton rats appeared to be targeted and sometimes “augmented” by SARS-CoV-2 infection. For example, “active” LDALT was more visible in some control, mock-inoculated SF than in SH, and LDALT was targeted by infection specifically in SF but not in SH. More abundant and potentially more “active” BM was detected at baseline in the head of naïve SH compared to SF, and BM was targeted more by SARS-CoV-2 infection in SH than in SF. Similarly, in the lung, the propensity to form thrombi and hyperplasia of blood vessel was seen in aged male SH and this tendency appeared to be increased by SARS-CoV-2 infection in this species. Hemosiderin-positive macrophages were seen most in young SH and their numbers increased after infection. These findings may indicate that biological predisposition to a certain process gets targeted by SARS-CoV-2 infection and, when dysregulated by infection, manifests in its augmented version. One potential mechanism of that effect would be through virus-mediated removal of some negative regulatory mechanism that keeps a particular predisposition in check. Biological processes in the body (including many diseases) are controlled through tightly regulated feedback mechanisms. Imbalance in that regulation may lead to augmentation of the above-mentioned processes and the development of severe diseases. Examples in this category may include autoimmune, hematologic/malignant disorders, as well as diabetes and neurological disorders. Dysregulation of these processes would be especially devastating for the elderly who have a high incidence of these disorders^61^. Coincidentally, all of these are complications of, as well as predisposing conditions of severe COVID in humans^62–64^. This may explain the high diversity of COVID manifestations in different human cohorts and highlight the need to dissect potential mechanisms in animal models.

## Materials and methods

### Animals

Inbred SH and SF cotton rats were obtained from a colony maintained at Sigmovir Biosystems, Inc. (Rockville, MD). Animals were housed in large polycarbonate cages and were fed a standard diet of rodent chow and water ad libitum. The colony was monitored for antibodies to paramyxoviruses and rodent viruses, and no such antibodies were found. The SARS-CoV-2 challenge studies were conducted by Sigmovir’s staff in the ABSL3 facility of Bioqual, Inc. upon completion of appropriate training administered by Bioqual’s personnel. Animals were transferred to Bioqual’s ABSL3 and allowed to acclimate before challenge with SARS-CoV-2. All studies were conducted under applicable laws and guidelines and after approval from both Sigmovir’s and Bioqual’s Animal Care and Use Committees.

### Virus

Two hCoV/USA-WA1/2020 SARS-CoV-2 preparations were used for the cotton rat challenge studies, with both viruses derived from the same seed virus (NRS-53721 BEI Resources).

The first study was run using SARS-CoV-2 HF-NR-53780, 1.58 × 10^6^ TCID_50_/ml or ~ 3.0 × 10^6^ PFU/ml, Genbank: MN985352.1, kindly provided by Clint Florence of NIH. The second study was run using Sigmovir’s stock of SARS-CoV-2 34C 120320 (1 × 10^6^ TCID_50_/ml), derived in Vero E6 cells from the same master seed NRS-53721. Viruses USA-WA1/2020 and USA-GNL-1205/2021 for neutralizing antibody assays were kindly provided by Adriana Kajon of Lovelace Biomedical Research Institute.

### Animal studies

#### SARS-CoV-2 challenge

Two independent studies using SARS-CoV-2 to challenge cotton rats SH and SF were conducted. In the first study (81 animals total), 24 young (2 months old) and 33 aged (> 9 months old) SH, as well as 24 medium-aged (5–6 months old) SF were inoculated with 10^5^ PFU/100 g SARS-CoV-2 intranasally (100 μl/100 g, distributed equally between both nostrils) and 2 × 10^4^ PFU/100 g ocularly (20 μl/100 g, 10 μl per eye, followed by eyelid rubbing) under isoflurane anesthesia. Starting from day 2 post-infection and until day 14 post-infection, blood and salivary swab samples were collected and animals were necropsied for organ collection at various time points after infection. Analysis of samples from this study for viral load provided the first indication of higher susceptibility of SF compared to SH to SARS-CoV-2 infection and guided design of the second cotton rat study. Lung histopathology samples from the first study were included in cumulative analysis of pulmonary pathology in both studies, with the medium-aged SF from the first study analyzed together with the young SF from the second study. In the second study (120 animals total), 28 young (2 months old) and 28 aged (8–9 months old) SH, and 28 young (3–4 months old) and 36 aged (8–10 months old) SF were used. Animals were infected intranasally and ocularly as described above and samples were collected starting from day 1 until day 16 post-infection. Sixteen infected animals (8 SF, 4 young and 4 aged, and 8 SH, 4 young and 4 aged, males and females) were re-challenged with SARS-CoV-2 16 days after the original infection and sacrificed 2 days later (“d18”). Nasal turbinates, lungs (left), and salivary swabs were collected for qPCR analysis. Right lung lobes were inflated in formalin for histopathology analysis. Heads (with lower jaw removed) were bisected sagittally, with one half immersed in formalin for histopathology and the other half used for extraction of turbinates for qPCR analysis. Males and females were included in each species/age category in equal numbers (where possible).

#### S protein vaccination

Eighteen five-month-old male and female SF were immunized with S protein expressed in mammalian expression system^65,66^ and adjuvanted with alum (2.5 μg S protein with 250 μg alum per animal), or only alum, 9 months before SARS-CoV-2 challenge. Animals were boosted with the same formulation 1, 2, and 3 months after the initial immunization. The time between the last boost and infection was 6 months.

#### Diabetic animals

Diabetes was induced in cotton rats via treatment with Streptozotocin. Four-to-five months old male and female cotton rats SF were treated intravenously with Streptozotocin at 50 mg/kg or inoculated intravenously with an equivalent volume of vehicle (0.01 M Sodium Citrate). A week later blood was collected for sugar quantification with a glucometer. Diabetic status in cotton rats was defined as ≥ 200 mg/dL in diabetic versus < 200 mg/dL blood sugar level in normal animals. Sixteen diabetic animals were infected with SARS-CoV-2 seven months after Streptozotocin treatment as described above and sacrificed on days 2 and 4 post-infection for viral load and histopathology analysis. Additional 4 animals were sacrificed on day 7 post-infection for analysis of serum neutralizing antibody response. The sustained diabetic status of animals included in the study was confirmed 1 week before SARS-CoV-2 challenge.

### Reverse-transcriptase polymerase chain reaction (RT-PCR) analysis

qPCR was run according to the protocol described previously^67^. In short, total RNA was extracted from homogenized turbinates and lung tissue using the RNeasy purification kit (QIAGEN) and from salivary swabs using Trizol. One μg of total RNA was used to prepare cDNA using Super Script II RT (Invitrogen) and oligo dT/random primers for total cDNA, or primers 5’-GGC AGA TTC CAA CGG TAC TAT TAC C-3’ and 5’-AGT GGC ACG TTG AGA AGA ATG TTA G-3’ for SARS-CoV-2-specific subgenomic cDNA. SARS-CoV-2 M gene was quantified in total and subgenomic cDNA using primers 5’-GGC TGT TAT GGC CAG TA ACTT TAG C-3’ and 5’GGA TTG AAT GAC ATG GAA CGC G-3’. The gene expression level in turbinates and lungs was normalized to the level of β-actin (“housekeeping gene”).

### Histopathology and immunohistochemistry

#### Lung

Lungs were prepared for histopathology analysis as previously described^68^. The presence of thrombi was evaluated for each lung sample as previously described^69^. Sections of lungs from the first cotton rat study were stained with Prussian Blue (Perl stain) for identification of hemosiderin-positive macrophages, which were quantified by a modified method of Golde^70^.

#### Nose

For nasal histopathology, heads were fixed in formalin, decalcified, and trimmed according to standard procedures^71^. Coronal sections posterior to the upper incisor teeth, incisive papilla, second palatal ridge, and upper first molar were generated and embedded in paraffin. Deparaffinized sections (5 μm) were stained with hematoxylin–eosin (H&E) or processed for immunohistochemistry (IHC). Antibody NR-52947 (BEI Resources, Polyclonal Anti-SARS-Related Coronavirus 2 Spike Glycoprotein IgG) and a goat anti-rabbit HRP-conjugated secondary antibody were used for IHC, followed by a DAB development and counterstaining with hematoxylin.

### Antibody assays

S-protein-binding IgG ELISA was run as previously described^65^. Titers of neutralizing antibodies to SARS-CoV-2 were determined in heat-inactivated cotton rat serum samples by micro-neutralization assay using Vero E6 and Vero E6 TMPRSS2 cells and SARS-CoV-2 stocks USA-WA1/2020 or USA-GNL-1205/2021 diluted to 100 TCID_50_/ 25 μL. Cells infected in the presence of serially-diluted sera were cultured for 3 days in DMEM, pen-step, 2% FBS at 37 °C, fixed with formalin, and stained with crystal violet. Fifty percent neutralization endpoints were calculated using the Reed and Muench method.

### Statistical analysis

ANOVA and t-test were used for statistical analysis, at least 4 cotton rats per time points (males and females) were included to account for experimental heterogeneity. Graphs were created using GraphPad Prism 9 or Excel; *p* value less than 0.05 was considered statistically significant for these studies.

### Ethical approval

*ARRIVE Guidelines*: The work is reported in accordance with ARRIVE guidelines (https://arriveguidelines.org).

## Supplementary Information

Supplementary Information.

## Data Availability

The authors confirm that the data supporting the findings of this study are available within the article and its Supplementary material. Raw data that support the findings of this study are available from the corresponding author, upon reasonable request.
