# Supplementary material for: SARS-CoV-2 infection augments species- and age-specific predispositions in cotton rats
Source: Sci Rep. 2023 Jan 14;13:757. doi: 10.1038/s41598-022-27328-y (PMC9840169; doi:10.1038/s41598-022-27328-y)
Supplement: Supplementary file 1 — Supplementary Information. [file 41598_2022_27328_MOESM1_ESM.docx]

**Supplementary information**

**SARS-CoV-2 Infection Augments Species- and Age-specific Predispositions in Cotton Rats**

Marina S. Boukhvalova, Emma Mortensen, Jessica Caple, John Joseph, Fatoumata Sylla, Arash Kamali, Daniel Stylos, Diego Lopez, Thomas March, Kevin M. Byrd, Gregory Prince, Ariel Arndt, Adriana Kajon, Jorge C.G. Blanco.

**Supplementary Table 1. Part 1**

|  | Time pi | Animal# | BM | LDALT | NALT | CT/ nerve |  |  | Time pi | Animal# | BM | LDALT | NALT | CT/ nerve |
| --- | --- | --- | --- | --- | --- | --- | --- | --- | --- | --- | --- | --- | --- | --- |
| **SF Young M** | PBS | 736 | - | - | ++ | + |  | **SF Aged M** | PBS | 529 | - | ++ |  |  |
|  |  | 737 | - | ++ |  |  |  |  |  | 530 | - | N | + |  |
|  | d1 | 720 | - | - | ++ |  |  |  | d1 | 531 | - | - | ++ |  |
|  |  | 721 | - | +++ | ++ |  |  |  |  | 532 | - | N | + |  |
|  | d2 | 722 | - | N | ++ |  |  |  | d2 | 533 | - | +++ | ++ |  |
|  |  | 723 | - | - |  |  |  |  |  | 534 | - | ++ | ++ |  |
|  | d4 | 724 | - | - | +++F |  |  |  |  | 438 | + | ++U,-L | ++ |  |
|  |  | 725 | - | N | + |  |  |  |  | 439 | ++ | ++U,NL | ++ | +++ |
|  | d7 | 726 | - | ++++ | +++ |  |  |  | d4 | 535 | N | N |  |  |
|  |  | 727 | - | N | + |  |  |  |  | 536 | - | - | +++ | + |
|  | d16 | 565 | - | +U,-L | +++ |  |  |  |  | 440 | - | ++ | ++ |  |
|  |  | 566 | - | +++ | +++ |  |  |  |  | 441 | - | ++++ | +++ |  |
|  | d16+2 | 567 | - | +++ | ++ |  |  |  | d7 | 537 | + | + | + |  |
|  |  | 568 | - | ++++ | +++ | ++ |  |  |  | 538 | - | N | + | +/- |
|  |  |  |  |  |  |  |  |  |  | 442 | - | ++++U,+L | + |  |
| **SF Young F** | PBS | 738 | - | - | + |  |  |  | d16 | 539 | - | - | ++F |  |
|  |  | 739 | - | - | ++ |  |  |  |  | 540 | - | ++ |  |  |
|  | d1 | 728 | - | +++ | + |  |  |  | d16+2 | 541 | - | - | ++F |  |
|  |  | 729 | - | +U,-L | +++ | +/- |  |  |  | 542 | - | ++ | ++F |  |
|  | d2 | 730 | - | - | ++ | +/- |  |  |  |  |  |  |  |  |
|  |  | 731 | - | - | ++ |  |  | **SF Aged F** | PBS | 543 | - | - | + |  |
|  | d4 | 732 | - | - | ++ | ++ |  |  |  | 544 | - | - |  |  |
|  |  | 733 | - | - |  |  |  |  | d1 | 545 | - | - |  |  |
|  | d7 | 734 | - | - | +++ |  |  |  |  | 546 | + | - |  | +++ |
|  |  | 735 | + | - | ++ |  |  |  | d2 | 547 | - | - | + |  |
|  | d16 | 569 | + | +++ | ++ | + |  |  |  | 548 | - | ++ | +++ |  |
|  |  | 570 | - | +++U,-L | +++F | + |  |  |  | 444 | + | ++ |  | ++ |
|  | d16+2 | 571 | + | ++++F | +++F |  |  |  |  | 445 | - | +++ | ++ | + |
|  |  | 572 | + | - | + | + |  |  | d4 | 549 | + | +++ | ++F | + |
|  |  |  |  |  |  |  |  |  |  | 550 | - | +++ | ++ |  |
|  |  |  |  |  |  |  |  |  |  | 446 | - | ++++F | ++ |  |
|  |  |  |  |  |  |  |  |  | d7 | 551 | + | ++ | +++F |  |
|  |  |  |  |  |  |  |  |  |  | 552 | - | +++ | +++F |  |
|  |  |  |  |  |  |  |  |  | d16 | 553 | +/- | +++ | ++ |  |
|  |  |  |  |  |  |  |  |  |  | 554 | - | - | ++ |  |
|  |  |  |  |  |  |  |  |  | d16+2 | 555 | + | +++F |  | + |
|  |  |  |  |  |  |  |  |  |  | 556 | - | ++ | ++ |  |

**Supplementary Table 1. Part 2.**

|  | Time pi | Animal# | BM | LDALT | NALT | CT/ nerve |  |  | Time pi | Animal number | BM | LDALT | NALT | CT/ nerve |
| --- | --- | --- | --- | --- | --- | --- | --- | --- | --- | --- | --- | --- | --- | --- |
| **SH Young M** | PBS | 3 | - | - |  |  |  | **SH Aged M** | PBS | 501 | - | - |  |  |
|  |  | 4 | - | - | + |  |  |  |  | 502 | - | - |  |  |
|  | d1 | 700 | - | - | + |  |  |  | d1 | 503 | + | N |  |  |
|  |  | 701 | - | - |  |  |  |  |  | 504 | - | - | +/- |  |
|  | d2 | 702 | + | - | + |  |  |  | d2 | 505 | - | N |  |  |
|  |  | 703 | ++ | - | + |  |  |  |  | 506 | + | - | + |  |
|  | d4 | 704 | + | - | + |  |  |  | d4 | 507 | ++ | - | + |  |
|  |  | 705 | ++ | + | + |  |  |  |  | 508 | ++ | N |  |  |
|  | d7 | 706 | ++ | - | +++ |  |  |  | d7 | 509 | + | - |  |  |
|  |  | 707 | +++ | - |  |  |  |  |  | 510 | - | - |  |  |
|  | d16 | 557 | + | - | +++ |  |  |  | d16 | 511 | - | - |  |  |
|  |  | 558 | + | - |  |  |  |  |  | 512 | - | - |  |  |
|  | d16+2 | 559 | - | - | + |  |  |  | d16+2 | 513 | + | - | + |  |
|  |  | 560 | + | - |  |  |  |  |  | 514 | + | +/- |  |  |
|  |  |  |  |  |  |  |  |  |  |  |  |  |  |  |
| **SH Young F** | PBS | 1 | + | - |  |  |  | **SH Aged F** | PBS | 515 | - | - |  |  |
|  |  | 2 | - | - |  |  |  |  |  | 516 | - | - |  |  |
|  | d1 | 708 | - | - | + |  |  |  | d1 | 517 | - | N |  |  |
|  |  | 709 | - | N | + |  |  |  |  | 518 | + | - | + |  |
|  | d2 | 710 | - | - | + |  |  |  | d2 | 519 | + | N |  |  |
|  |  | 711 | + | - | + |  |  |  |  | 520 | ++ | - |  |  |
|  | d4 | 712 | - | - | + |  |  |  | d4 | 521 | + | - |  |  |
|  |  | 713 | - | - |  |  |  |  |  | 522 | + | - |  |  |
|  | d7 | 714 | + | - | + |  |  |  | d7 | 523 | + | N | + |  |
|  |  | 715 | + | +U,-L | ++ | + |  |  |  | 524 | ++ | - | ++ | + |
|  | d16 | 561 | - | - | ++ |  |  |  | d16 | 525 | + | - |  |  |
|  |  | 562 | + | - | ++ |  |  |  |  | 526 | ++ | + |  |  |
|  | d16+2 | 563 | - | - |  |  |  |  | d16+2 | 527 | + | - | + |  |
|  |  | 564 | + | N |  |  |  |  |  | 528 | ++ | - |  |  |

**Supplementary Table 1**. Histological changes in the paranasal/paraoral tissues of SARS-CoV-2-infected cotton rats SF (Part I) and SH (Part II). Animals were infected with SARS-CoV-2 as described in the legend to Fig. 1. Heads were bisected (after removal of the lower jaw), with one half fixed in formalin, decalcified, embedded in paraffin, and stained with H&E. Bone marrow hyperplasia (BM), hyperplasia of Lacrimal Drainage-associated Lymphoid Tissue (LDALT) or Nasal-Associated Lymphoid Tissue (NALT), and inflammation in the connective tissue in the vicinity of infraorbital nerve and tooth root (CT/nerve) were evaluated. N, structure/area not visible. Notations referring to LDALT: L, “lower” (rostral) portion of nasolacrimal duct (NLD); U, upper (caudal) portion of NLD. Caudal portion of NLD was visible only in a few samples in this study and is marked where detected (the rest of the masurements are representative of the rostral portion of NLD). Shaded in pink: “occlusion” of osseous canal housing NLD. F, follicular-type germinal center. Changes in NALT are indicated only for the samples where presence of NALT was identifiable. Changes in CT/nerve are indicated only for samples in which inflammation in the area was detected. Representative results from the second challenge study are shown. Data for young males and females (Young M and Young F) and aged males and females (Aged M and Aged F) for each species of cotton rats is shown separately.

|  | **Study 1** | |  | **Study 2** | |  | **Cumulative** | |
| --- | --- | --- | --- | --- | --- | --- | --- | --- |
| **Day pi** | **Males** | **Females** |  | **Males** | **Females** |  | **Males** | **Females** |
| **0** | 0/4 | 0/4 |  | 1/2 | 0/2 |  | 1/6 (17%) | 0/6 (0%) |
| **1** | NA | NA |  | 2/2 | 0/2 |  | 2/2 (100%) | 0/2 (0%) |
| **2** | 2/3 | 0/3 |  | 1/2 | 0/2 |  | 3/5 (60%) | 0/5 (0%) |
| **4** | 1/3 | 0/4 |  | 1/2 | 0/2 |  | 2/5 (40%) | 0/5 (0%) |
| **7** | 0/3 | 0/5 |  | 0/2 | 1/2 |  | 0/5 (0%) | 1/5 (20%) |
| **14** | 1/3 | 0/6 |  | NA | NA |  | 1/3 (33%) | 0/3 (0%) |
| **16** | NA | NA |  | 1/2 | 0/2 |  | 1/2 (50%) | 0/2 (0%) |
| **18** | NA | NA |  | 2/2 | 0/2 |  | 2/2 (100%) | 0/2 (0%) |

**Supplementary Table 2**. Pulmonary thrombosis in aged cotton rats SH infected with SARS-CoV-2. Animals were challenged with SARS-CoV-2 as described in the legend to Fig. 1. Lungs were collected on different days post-infection, stained with H&E, and evaluated for the presence of thrombi in medium to large size blood vessels (photomicrographic examples are shown in Fig. 6B). Data is presented for males (columns shaded in grey) and females SH in studies 1 and 2 separately, and cumulatively. Number of thrombi-positive animal per group is shown for each study, and total percentage of thrombi-positive samples is given for the cumulative data (in parenthesis). NA: sample was not available for evaluation in that particular study.
